# Trait Emotional Intelligence Profiles of Parents With Drug Addiction and of Their Offspring

**DOI:** 10.3389/fpsyg.2018.01633

**Published:** 2018-09-05

**Authors:** Georgia S. Aslanidou, K. V. Petrides, Ariadni Stogiannidou

**Affiliations:** ^1^Aristotle University of Thessaloniki, Thessaloniki, Greece; ^2^University College London, London, United Kingdom

**Keywords:** addiction, substance abuse, mental health, TEIQue, children of parents with drug addiction

## Abstract

This study examines the relationship between trait emotional intelligence (trait EI) and general health (General Health; GHQ-28) in two samples of Greek parents with (*n* = 52; Mage = 39.78; SDage = 6.68; 41 men and 11 women) and without (*n* = 51; Mage = 43.53; SDage = 4.61; 40 men and 11 women) addiction problems. In addition, it compares the trait EI scores of their offspring (*N* = 81; Mage = 11.71; SDage = 2.15; 51 boys and 30 girls). Results showed that parents with drug addiction exhibited lower levels of trait EI and poorer general health than peers. In addition, global trait EI and two of its subscales, Well-being and Emotionality, had stronger correlations with depression in the addiction than in the comparison group. Well-being was a significant predictor of general health and its subscales (Somatic symptoms, Anxiety/insomnia, Social dysfunction, and severe depression) in both groups. No differences were found between the offspring of the two groups.

## Introduction

### Emotions and Addiction

The American Psychiatric Association (2013) summarizes the required criteria for the diagnosis of substance use disorders in four broad categories: difficulties in controlling substance consumption; an impact on the individual’s social life; substance use despite the physical or psychological harm; and pharmacological components, such as withdrawal and tolerance. The term addiction, although not included in the APA manual, is often used in order to describe the established and severe substance use (O’Brien, 2011; American Psychiatric Association, 2013).

Comorbidity of addiction with mental and affective disorders is quite high and has received a lot of attention in the literature (Bennett, 2002; Kassel and Veilleux, 2010; Lai et al., 2015). Affective disorders, such as depression, are especially common in opiates use (Matsa, 2006; Compton et al., 2007; American Psychiatric Association, 2013). The occurrence of both affective and substance use disorders can exacerbate the impact of the symptoms on well-being, relating to others, and everyday functioning. It also highlights the connection between substance use and emotion regulation difficulties (Bennett, 2002; Witkiewitz and Wu, 2010).

Individuals addicted to drugs are overwhelmed by their feelings. They have difficulties in differentiating and expressing emotions, especially negative ones (Baker et al., 2004). Substances are often used as a means of regulating pain, stress or frustration (Freud, 1930; Matsa, 2008; Witkiewitz and Wu, 2010), a possibility also proposed by Khantzian (1985, 1997) in his “self-medication hypothesis.” As further discussed below, this hypothesis is particularly interesting from the perspective of trait emotional intelligence (trait EI) theory because it suggests that optimization of trait EI profiles as a means of emotion regulation could reduce the need for reliance on drug use. Indeed, such optimization has also been suggested in relation to medically approved pharmacological interventions in order to tackle recognized psychopathological conditions (Petrides et al., 2017).

### Trait Emotional Intelligence

Trait emotional intelligence (trait EI or trait emotional self-efficacy) refers to a constellation of emotional self-perceptions assessed by questionnaires and rating scales (Petrides et al., 2007). Essentially, the construct concerns people’s perceptions of their emotional abilities.

Trait EI has been shown to predict a variety of life outcomes, such as psychological resilience, socioemotional competence, and peer relations (Frederickson et al., 2012). Its relationship with health has been demonstrated in many studies (Costa et al., 2014), including a large meta-analysis covering mental, psychosomatic, and physical health (Martins et al., 2010; see also Andrei et al., 2016). However, in contrast to trait EI links with mental health, the construct’s links with addiction and substance use have received very limited attention (Kun and Demetrovics, 2010). This represents a clear gap in the literature, particularly when we take into account the central role of emotions in addictive behaviors.

### Personality and Addiction

Individuals with drug addiction experience difficulties in the accurate evaluation of emotional cues and in appreciating the consequences of their actions to themselves and to other people (Khantzian, 1997; Kornreich et al., 2003; McCrady and Epstein, 2013). Terracciano et al. (2008) examined the profiles of drug users on the Big Five personality dimensions. They found that heroin and cocaine users scored higher on Neuroticism, were more susceptible to depressive feelings and hostility, more impulsive, more easily stressed out, and more likely to feel guilty and ashamed than all other participating groups (tobacco smokers, marijuana users, and a comparison group). In addition, they scored lowest on the major personality dimension of Conscientiousness as well as on various socially desirable facets, like excitement seeking (Extraversion) and straightforwardness, trust, and compliance (Agreeableness).

A study conducted in Norway with individuals addicted to opioids yielded similar results (Kornør and Nordvik, 2007). Generally, Neuroticism was elevated in individuals with drug addiction relative to controls even when different personality tests were employed. However, Brooner et al. (2002) noted that when potentially confounding factors like mental disorders were controlled for, the personality traits most robustly differentiating people with opioid addiction from non-addicted peers were Conscientiousness, Agreeableness, and Excitement-seeking. In addition, Sutin et al. (2013) examined the role of poverty in the relationships between personality traits and drugs. They concluded that high neuroticism and low agreeableness were related to drug use regardless of poverty status. In contrast, low Conscientiousness proved to be a risk factor only under specific financial conditions.

### Emotional Intelligence and Addiction

Kun and Demetrovics (2010) traced the relationship between EI and related constructs with addictions back from 1990 until 2009 and found that low EI was associated with more addiction problems. In terms of EI components, “decoding and differentiation of emotions” and “regulation of emotions” seemed to contribute significantly to addictions. Considering the limitations of previous research, Kun and Demetrovics (2010) pointed out the need for conducting separate studies, based on the type of the substance abused (heroin, cocaine, etc.), and proposed that samples be derived from clinical, instead of community, populations. With respect to trait EI specifically, negative relationships have been reported in both adults and adolescents (Riley and Schutte, 2003; Resurrección et al., 2014).

While the relationship between trait EI and addiction is under-researched, that between parental addiction and offspring trait EI has never been addressed. Moreover, no studies have compared the trait EI profiles of children of parents with versus without addiction. Some researchers (e.g., Scaife, 2007), argued that the children of parents with addiction exhibit emotional difficulties and may be at greater risk of becoming involved with drugs. However, others claim that most such children can function satisfactorily in the emotional and behavioral domains despite the adverse circumstances (e.g., Pilowsky et al., 2004; Velleman and Templeton, 2016).

### The Present Study

In consideration of the call for clinical and substance-specific samples in research on trait EI and addiction (Kun and Demetrovics, 2010), we decided to study participants in an opiate substitution therapy program. Because we were interested in the trait EI profiles of both parents and their children, eligibility to the study was restricted to individuals with addiction who were parents. Since childhood and adolescence are developmentally different periods (Shaffer and Kipp, 2013), children and adolescents were examined separately.

The first objective of the study was to examine if individuals with drug addiction differ from peers in trait EI, general health, and their various subscales (well-being, self-control, emotionality, and sociability for trait EI; somatic symptoms, anxiety-insomnia, social dysfunction, and severe depression for general health). In line with previous research (Riley and Schutte, 2003; Kun and Demetrovics, 2010; Resurrección et al., 2014), it was hypothesized that individuals with drug addiction would score lower on trait EI and general health and their subscales than a comparison group. Parents with drug addiction are characterized by lack of emotionality and control, conflicted relationships, and insecure parent-child attachment relationships (Scaife, 2007). Accordingly, it was hypothesized that the offspring of parents with drug addiction would score lower on trait EI than the comparison group. It should be noted here that, to the best of our knowledge, this is the first study to investigate trait EI in the children of parents with drug addiction. Considering the connection between trait EI and life outcomes, such as academic performance and psychosocial adaptation (Mavroveli et al., 2009; Ferrando et al., 2011), the identification of low trait EI in children of parents with drug addiction could well suggest an area of early intervention.

The current research design incorporates a comparison group as well as indicators of mental health. Accordingly, associations between trait EI and general health are explored both in the addiction and in the comparison group. Based on trait EI theory, which predicts that emotions are fundamentally important to health (see Petrides et al., 2016) and given that individuals with addiction experience severe emotional disturbances (Lai et al., 2015), stronger correlations between trait EI and general health were anticipated in the addiction than the comparison group.

Correlations between parent-offspring trait EI scores were examined, separately in the addiction and comparison groups. Based on previous research (e.g., Vernon et al., 2008; Gugliandolo et al., 2015) we expected these correlations to be moderately strong.

In summary, the hypotheses of the study were as follows:

- *Hypothesis 1*: Individuals with drug addiction would score lower than their comparison group peers on trait EI and general health and their subscales.
- *Hypothesis 2*: Offspring of parents with drug addiction would score lower on trait EI than their peers in the comparison group.
- *Hypothesis 3*: Correlations between trait EI and general health would be stronger in the addiction than the comparison group.
- *Hypothesis 4*: There would be significant correlations between parent-offspring trait EI scores in both the addiction and the comparison groups.

In addition to these hypotheses, which mostly concerned global trait EI scores, for purposes of completeness and in order to identify the source of any differences in global trait EI scores we extended the analysis to the factor level of the construct (Well-being, Self-control, Emotionality, and Sociability) without, however, advancing specific hypotheses about these results.

## Materials and Methods

### Participants

The sample comprised 103 adult participants, 42 adolescents and 39 children (see **Table 1** for detailed descriptives). Among the adults, 52 were addicted to drugs and were recruited from substitution units of the Organization Against Drugs Program in Thessaloniki, Greece. At the time the research was conducted, five units existed in Thessaloniki all of which were approached. Comparison group participants were recruited from three primary and two secondary schools and were carefully matched to the addiction group on parental gender and nationality as well as on offspring age and gender. Schools were selected from regions that represented the socioeconomic status of the participants in the experimental group as closely as possible.

**Table 1:** Participant age statistics for the addiction and comparison groups.

Group	Addiction			Comparison		
	M	SD	N	M	SD	N
Parent	39.78	6.68	52	43.53	4.61	51
Father	39.76	7.21	41	43.78	4.73	40
Mother	39.86	4.38	11	42.62	4.21	11
Adolescent	13.40	1.12	20	13.54	0.90	22
Boy	13.61	1.00	14	13.50	1.04	15
Girl	12.90	1.31	6	13.62	0.51	7
Child	9.79	1.27	19	9.85	1.24	20
Boy	9.69	1.49	11	9.59	1.13	11
Girl	9.93	0.98	8	10.16	1.37	9


Parents of children younger than eight or older than fifteen and a half years were excluded from the study. This age span was chosen firstly because research on offspring of parents with drug addiction has hitherto focused mainly on the early or late developmental stages and less on the middle period, and secondly, because the TEIQue-CSF cannot be used with children younger than 8 years old. Parents with serious disorders like severe depression, psychosis, etc., were also excluded. In order for severe disorders to be identified, diagnostic records were checked, oral reports from therapists were obtained and, in some cases, a short interview was conducted by a researcher. From the fifty-two parents who participated in the study, 20 adolescents and 19 children completed the appropriate TEIQue instruments. The remaining 13 declined to participate.

### Measures

*Trait Emotional Intelligence Questionnaires* (TEIQue; Petrides, 2009). This family of instruments has been designed to assess the affective aspects of personality that are comprehensively encompassed by trait EI in various populations. In the present study, the relevant Greek adaptations (Petrides et al., 2007; Stamatopoulou et al., 2016) were used.

Parents completed the TEIQue-SF (Cooper and Petrides, 2010), which comprises 30 items and yields scores on global trait EI and its four subscales (Well-being, Self-control, Emotionality, and Sociability). Adolescents filled out the 30-item adolescent short form (TEIQue-ASF; Petrides et al., 2006). Finally, children completed the TEIQue-CSF (Mavroveli et al., 2008), which comprises 36 items and yields a global trait EI score only. Internal consistencies for all TEIQue scores are presented in **Table 2**.

**Table 2:** Internal consistencies for the TEIQue-SF and GHQ variables.

Scale	Total sample	Addiction group	Comparison group
Parent			
Global trait EI	0.78	0.77	0.78
Well-being	0.61	0.62	0.60
Self-control	0.61	0.56	0.66
Emotionality	0.50	0.41	0.59
Sociability	0.50	0.42	0.54
GHQ Global score	0.92	0.92	0.90
Somatic symptoms	0.79	0.79	0.74
Anxiety – insomnia	0.83	0.77	0.81
Social dysfunction	0.79	0.81	0.76
Severe depression	0.86	0.86	0.76
Adolescent			
Global trait EI	0.87	0.90	0.78
Children			
Global trait EI	0.81	0.86	0.68


*EI, emotional intelligence; GHQ, General Health Questionnaire.*

*General Health Questionnaire* (GHQ-28; Goldberg, 1978; Moutzoukis et al., 1990). The GHQ assesses mental, physical, and emotional well-being. Parents completed the 28-item version, comprising four 7-item subscales (somatic symptoms, anxiety-insomnia, social dysfunction, and severe depression). Higher scores on GHQ actually indicate poorer health. Internal consistencies for the GHQ variables are presented in **Table 2**.

### Procedure

Participants were approached through an opiate substitution program operating in Greece, as mentioned above. This allowed us to control for type (opiates) and severity of drug use (participants in substitution programs all suffer from severe addiction). Participants were admitted on the basis that addiction in opiates was fully established. This was certified by extensive screening tests and interviews, based on the DSM criteria, and conducted by a psychiatrist, a psychologist, and a social worker. Opiate addiction did not preclude the use of other substances, but it did mean that opiates were both the drug for which treatment was sought and the primary drug of use as shown by regular urine tests conducted during the treatment program. Authorized substances for opiate substitution treatment were methadone and buprenorphine.

Members of the units who were parents were identified from unit records. Parents were approached by a researcher and asked if they wished to participate in the study. In the second part of the data collection, parents were recruited for the comparison group. Researchers visited three primary and two secondary schools and administered the materials to children at the beginning of class. One envelope included the parent’s questionnaires, with a brief description of the study, and the second the child’s questionnaires. Pupils were asked to return the questionnaires to their teachers the following day.

Participants were assured that their answers would remain strictly confidential. They were informed that they had the right to withdraw at any time, if they wished to do so, and that they were free to not answer any questions that made them feel uncomfortable. They were also instructed that there were no right or wrong responses to the questionnaires. Informed consent for children and adolescents was obtained by parents or guardians. For the comparison group, written permission was also obtained from the Greek Ministry of Education, Lifelong Learning, and Religious affairs.

## Results

Chi-square tests showed no significant differences between the two groups (addiction and comparison) in terms of parental gender, *χ*^2^(1) = 0.003, *p* = 0.96 and nationality, *χ*^2^(3) = 1.87, *p* = 0.60. Similarly, there were no differences in terms of number of children, *χ*^2^(1) = 0.03, *p* = 0.86, and adolescent gender, *χ*^2^(1) = 0.02, *p* = 0.90. *T*-tests showed that children, *t*(37) = 0.14, *p* = 0.89 and adolescents, *t*(40) = 0.46, *p* = 0.65 did not differ in age either.

However, there were some significant differences between the two groups. Thus, mean educational level was lower in the addiction (*M* = 2.23, SD = 0.96) than the comparison (*M* = 3.39, SD = 1.36) group, *t*(101) = 5.02; *p* < 0.01. In the group with addiction, the percentage of participants who had gone beyond compulsory education (primary and secondary school) was 34.6%, whereas in the comparison group, it was 82.3%. The mean educational level of the partners (M_addiction_ = 2.80, SD_addiction_ = 1.26; M_comparison_ = 3.16, SD_comparison_ = 1.03) was similar for both groups [*t*(97) = 1.60; *p* = 0.12]. Last, a *t*-test and a chi-square test, respectively, showed that the addiction and comparison groups differed in average parental age *t*(90.76) = 3.32, *p* < 0.01 (see **Table 1**) and marital status, *χ*^2^(4) = 15.62, *p* < 0.01. In the addiction group, 55.8% of participants were married, 19.2% divorced, 7.7% had a single-parent family, 13.5% lived together with a partner, and 3.8% were widowed. In the comparison group, 88.2% of the participants were married, 9.8% divorced and 2% had a single-parent family.

There were no gender differences in trait EI [*t*(97) = 0.90; *p* = 0.37] or general health [*t*(101) = 1.15; *p* = 0.25], thus the data were combined in subsequent analyses.

One-way ANOVAs with addiction status as the independent variable (addiction vs. comparison group) and global trait EI, general health and their subscales as the dependent variables were performed to test hypothesis H1. Similarly, one-way ANOVAs with addiction status as the independent variable and children and adolescents’ trait EI as the dependent variables were used to test hypothesis H2.

The foregoing results are summarized in **Table 3**, where it can be seen that parents in the group with addiction scored lower on trait EI and higher on general health (indicating that they experienced *poorer* health) than parents in the comparison group. The only subscales that did not follow this pattern were emotionality (from trait EI) and Social dysfunction (from general health). These results supported H1, but not H2, since there were no differences in the scores of offspring.

**Table 3:** Means, SD, and one-way analyses of variance (ANOVA) for the effects of addiction on trait EI and general health.

	Addiction		Comparison		ANOVA	
Variable	M	SD	M	SD	F	Partial η^2^
Parent (*n* = 103)						
Global trait EI	4.68	0.70	5.04	0.65	6.80^†^	0.07
Well-being	4.93	1.08	5.52	1.01	8.23*	0.08
Self-control	4.36	1.13	4.81	1.15	3.83^†^	0.04
Emotionality	4.79	0.85	4.91	0.90	0.51	0.01
Sociability	4.28	1.03	4.76	0.93	6.19^†^	0.06
GHQ Global score	26.92	14.85	18.02	9.71	12.92*	0.11
Somatic symptoms	6.75	4.29	4.20	2.88	12.53*	0.11
Anxiety – insomnia	9.38	4.54	4.84	3.70	30.91**	0.23
Social dysfunction	5.98	4.16	7.20	2.91	2.94	0.03
Severe depression	4.81	4.93	1.78	2.42	15.51**	0.13
Adolescent (*n* = 42)						
Global trait EI	4.74	0.62	4.92	0.61	0.84	0.02
Child (*n* = 39)						
Global trait EI	3.73	0.38	3.72	0.33	0.01	0.00


*EI, emotional intelligence; GHQ, General Health Questionnaire. Degrees of freedom for the denominator ranged between 97 and 101 for adults and were 40 for adolescents and 37 for children. η^2^ = effect size.*

^†^*p < 0.05, one-tailed. ^∗^p < 0.05, two-tailed. ^∗∗^p < 0.01, two-tailed.*

In order to investigate whether the trait EI – general health relationships are stronger in the addiction than the comparison group (hypothesis H3), Pearson product-moment correlations were calculated (see **Table 4**) and compared statistically (see **Table 5**^1^).

**Table 4:** Correlations between global trait EI, general health, and their subscales for parents in the addiction (*n* = 52; above the diagonal) and comparison (*n* = 51; below the diagonal) groups.

	1	2	3	4	5	6	7	8	9	10
1. Trait EI	–	0.62**	0.71**	0.73**	0.63**	–0.43**	–0.30*	–0.25	–0.29*	–0.55**
2. Well-being	0.61**	–	0.27	0.23	0.18	–0.56**	–0.42**	–0.40**	–0.27	–0.67**
3. Self-control	0.73**	0.37**	–	0.40**	0.33*	–0.29*	–0.10	–0.27	–0.20	–0.37**
4. Emotionality	0.75**	0.24	0.38**	–	0.30*	–0.19	–0.11	–0.04	–0.14	–0.30*
5. Sociability	0.47**	0.10	0.20	0.19	–	–0.26	–0.23	–0.14	–0.24	–0.23
6. General health	–0.27	–0.51**	–0.31*	–0.03	0.26	–	0.87**	0.80**	0.78**	0.87**
7. Somatic symptoms	–0.12	–0.39**	–0.13	0.01	0.38**	0.84**	–	0.61**	0.60**	0.67**
8. Anxiety – insomnia	–0.26	–0.50**	–0.27	–0.04	0.28	0.88**	0.69**	–	0.41**	0.59**
9. Social dysfunction	–0.24	–0.40**	–0.21	–0.09	0.14	0.79**	0.60**	0.55**	–	0.59**
10. Severe depression	–0.27	–0.32*	–0.44**	0.04	–0.01	0.70**	0.41**	0.54**	0.42**	–


*EI, emotional intelligence.*

*^∗^p < 0.05, two-tailed. ^∗∗^p < 0.01, two-tailed.*

**Table 5:** Statistical comparisons (Fisher’s z) of the correlations between trait EI, general health, and their subscales for parents in the addiction and comparison groups.

	Addiction	Comparison	*Z*
Well-being – GHQ	–0.56**	–0.51**	0.34
Well-being – somatic symptoms	–0.42**	–0.39**	0.18
Well-being – anxiety – insomnia	–0.40**	–0.50**	–0.62
Well-being – severe depression	–0.67**	–0.32*	2.35**
Self-control – GHQ	–0.29*	–0.31*	–0.11
Self-control – severe depression	–0.37**	–0.44**	–0.41


*EI, emotional intelligence; GHQ, General Health Questionnaire.*

^∗^*p < 0.05, two-tailed. ^∗∗^p < 0.01, two-tailed.*

High correlations between trait EI and its subscales, and general health and its subscales were immediately apparent in both groups. In the parents with addiction group, trait EI correlated negatively with general health (*r* = -0.43, *p* < 0.01) and its subscales, with the exception of anxiety-insomnia. The strongest correlation was observed between trait EI and Depression (*r* = -0.55, *p* < 0.01). In contrast, trait EI and general health were uncorrelated in the comparison group.

Among all trait EI subscales, Well-being exhibited the strongest correlations with general health and its subscales. Results were quite similar across both groups. However, in the addiction group, Well-being did not correlate significantly with Social dysfunction, while it correlated with Depression more strongly than in the comparison group (*r_ag_* = -0.67, *p* < 0.01; *r_cg_* = -0.32, *p* < 0.05; Fisher *z* = 2.36, *p* < 0.05). Self-control correlated negatively with general health (*r_ag_* = -0.29, *p* < 0.05; *r_cg_* = -0.31, *p* < 0.05) and Depression (*r_ag_* = -0.37, *p* < 0.01; *r_cg_* = -0.44, *p* < 0.01) in both groups (see **Table 5**). With the sole exception of a negative correlation with Depression in the addicted group (*r* = -0.30, *p* < 0.05), the Emotionality subscale of trait EI was not significantly correlated with general health or its subscales. Similarly, Sociability did not correlate with general health or its subscales, apart from somatic symptoms in the comparison group (*r* = 0.38, *p* < 0.01).

To determine the strongest predictors of general health, we performed a series of regressions with the total score and its subscales as DVs and the trait EI subscales as IVs, separately in individuals with addiction and peers. Inspection of VIF values showed no evidence of multicollinearity. Specifically, in the addiction group values ranged from 1.11 to 1.30 and in the comparison group from 1.06 to 1.31. All regressions reached statistical significance, except for Social dysfunction in the addiction group. Detailed results are presented in **Tables 6** (addiction group) and **7** (comparison group). It can be seen there that, in the group with addiction, the Well-being subscale of trait EI was the best predictor of general health and its subscales, with its strongest influence on Depression (the relationships were negative because higher scores on the GHQ-28 indicate poorer health). Findings were less consistent in the comparison group. More specifically: (i) Well-being was negatively related to (poor) general health and three of its subscales, (ii) Sociability was positively related to (poor) general health and its somatic symptoms subscale and negatively to the anxiety-insomnia subscale, and (iii) Self-control was negatively related to the Depression subscale.

**Table 6:** Regressions of general health and its subscales on the four trait EI subscales in the addiction group.

Dependent variable	R	R^2^	Adjusted R^2^	F (df)	TEIQue	β	*t*
General health	0.607	0.368	0.309	6.26^∗∗^ (4, 43)	Wb	–0.56	4.37**
					Sc	–0.01	0.10
					Em	–0.01	0.11
					So	–0.13	1.01
Somatic symptoms	0.490	0.241	0.170	3.41^∗^ (4, 43)	Wb	–0.44	3.12**
					Sc	0.16	1.05
					Em	0.01	0.02
					So	–0.23	1.56
Anxiety – insomnia	0.454	0.206	0.132	2.79^∗^ (4, 43)	Wb	–0.42	2.90**
					Sc	–0.14	0.87
					Em	0.11	0.75
					So	–0.03	0.19
Social dysfunction	0.343	0.118	0.036	1.44 (4, 43)	Wb	–0.27	1.80
					Sc	0.03	0.16
					Em	–0.06	0.36
					So	–0.14	0.92
Severe depression	0.720	0.518	0.473	11.56^∗∗^ (4, 43)	Wb	–0.65	5.78*
					Sc	–0.08	0.63
					Em	–0.10	0.87
					So	–0.05	0.43


*Wb, well-being; Sc, self-control; Em, emotionality; So, sociability.*

^∗^*p < 0.05, two-tailed. ^∗∗^p < 0.01, two-tailed.*

**Table 7:** Regressions of general health and its subscales on the four trait EI subscales in the comparison group.

Dependent variable	R	R^2^	Adjusted R^2^	F (df)	TEIQue	β	*t*
General health	0.631	0.398	0.346	7.60^∗∗^ (4, 46)	Wb	–0.48	3.85**
					Sc	–0.24	1.86
					Em	0.11	0.88
					So	0.33	2.81**
Somatic symptoms	0.579	0.335	0.277	5.80^∗∗^ (4, 46)	Wb	–0.42	3.22**
					Sc	–0.08	0.58
					Em	0.05	0.37
					So	0.43	3.44**
Anxiety – insomnia	0.613	0.376	0.321	6.92^∗∗^ (4, 46)	Wb	–0.48	3.81**
					Sc	–0.19	1.41
					Em	0.08	0.63
					So	–0.34	2.82**
Social dysfunction	0.449	0.201	0.132	2.90^∗^ (4, 46)	Wb	–0.38	2.66*
					Sc	–0.11	0.73
					Em	0.01	0.05
					So	0.20	1.48
Severe depression	0.533	0.285	0.222	4.57^∗∗^ (4, 46)	Wb	–0.22	1.63
					Sc	–0.47	3.26*
					Em	0.26	1.86
					So	0.06	0.49


*Wb, well-being; Sc, self-control; Em, emotionality; So, sociability.*

^∗^*p < 0.05, two-tailed. ^∗∗^p < 0.01, two-tailed.*

In order to test hypothesis (H4), parent–offspring correlations were computed in the total sample and in the two groups separately (**Table 8**). Although the trends were in the expected direction, results showed no significant relationships for adolescent trait EI in either of the two groups (addiction and non-addiction) or the total sample. Findings were quite similar for children, whose global trait EI scores correlated significantly only with (i) parental global trait EI and Self-control in the total sample and (ii) parental Self-control in the addiction group.

**Table 8:** Correlations between parental variables (in rows) and adolescent and children’s global trait EI in the total, addiction, and comparison samples.

	Adolescent global trait EI			Children global trait EI		
	Total sample (*N* = 42)	Addiction (*n* = 20)	Comparison (*n* = 22)	Total sample (*N* = 39)	Addiction (*n* = 19)	Comparison (*n* = 20)
Global trait EI	0.18	0.33	0.27	0.40*	0.49	0.28
Well-being	0.21	0.36	0.28	–0.03	–0.09	0.01
Self-control	0.20	0.30	0.36	0.33*	0.55*	0.07
Emotionality	0.06	0.17	0.18	0.19	0.10	0.25
Sociability	0.13	0.21	0.38	0.14	0.05	0.20
General health	–0.01	–0.19	0.02	0.04	0.08	0.03
Somatic symptoms	0.01	–0.13	–0.04	–0.02	–0.08	0.18
Anxiety – insomnia	0.02	–0.10	0.23	–0.05	0.01	–0.02
Social dysfunction	–0.04	–0.16	–0.06	0.17	0.19	–0.07
Severe depression	–0.04	–0.15	0.03	0.07	0.14	0.06
Age of parents	–0.03	0.21	–0.17	0.15	0.28	–0.18
Education of parents	0.08	0.08	0.02	0.08	–0.05	0.20


*^∗^p < 0.05, two-tailed.*

## Discussion

### Trait EI, General Health and Parental Addiction

One important aim of our study was to examine if parents with drug addiction and their children differ in their trait EI profiles from their addiction-free peers. As hypothesized (H1), apart from the Emotionality subscale, parents in the addiction group scored lower on trait EI than parents in the comparison group. This is in accordance with previous research revealing various impairments in the emotional attributes of people with drug addiction (Schutte et al., 1998; Kornreich et al., 2003; Kun and Demetrovics, 2010). In addition, people with drug addiction tend to have restricted social networks. They are either isolated or socialize mainly with other people with drug addiction (Sherman et al., 2002; Scaife, 2007; Livingston et al., 2012). Communication in these relationships is not authentic, revolving around the substance of use, and obstructing the processing and sharing of feelings (McIntosh and McKeganey, 2000).

As regards general health, the literature supports our finding that depression and anxiety symptomatology is higher in people with addiction than in controls (Merikangas et al., 1998; Compton et al., 2007; Lai et al., 2015). Individuals with drug addiction often report negative feelings or conditions like sadness, hopelessness, stress, and insomnia. Somatic symptoms are also elevated in populations with drug addiction. Participants in treatment programs usually have a history of years of addiction. Their health condition is compromised due to poor standards of hygiene, neglect of health problems, and intravenous use, while contagious diseases, like hepatitis C, are common (Backmund et al., 2005; Friedman et al., 2006; Nelson et al., 2011). Accordingly, we expected individuals addicted to drugs to report more somatic symptoms than the comparison group. Social dysfunction was the only subscale of general health that did not differ between the two groups. It could be that participation in a treatment program enhanced the sense of self-efficacy in individuals with drug addiction as well as their perceived ability to manage routines and daily practice, although this finding should ideally be replicated on a new sample.

Hypothesis H3 was partially borne out by the data, as trait EI and one of its subscales correlated significantly more strongly with depression in the drug-addiction than the comparison group. Emotions are of central relevance to health and the overrepresentation of affective disorders in populations with drug addiction (Merikangas et al., 1998) means that trait EI will be even more important to such populations than to typical peers. As has been shown (e.g., Espinosa and Rudenstine, 2018; Rudenstine and Espinosa, 2018), trait EI can contribute to the treatment of depression, anxiety and borderline personality disorders related to life trauma (see also Nelis et al., 2011). Higher trait EI may also play a protective role in relation to mental health symptomatology (Lea et al., 2018). It follows, then, that training in trait EI could contribute to the reduction of drug addiction difficulties.

Global trait EI did not correlate significantly with symptoms of anxiety-insomnia in the drug-addiction group, although its Well-being subscale did, as expected. Contrary to previous research (Martins et al., 2010), global trait EI was not significantly correlated with general health or its subscales in the comparison group. However, this was probably an artifact of the small sample size, since a clear pattern of negative relationships was observed, as expected. Moreover, the Well-being subscale of trait EI was significantly negatively correlated with general health and all of its subscales. Despite differences of magnitude, Well-being had a unique effect on general health and its subscales in both the addiction and the comparison groups. Three differences in the results of individuals with addiction and those without were most notable. First, in the comparison group, the Self-control subscale of trait EI explained most of the variance in Depression, whereas in the addiction group the strongest predictor was the Well-being subscale. For non-addicted individuals, Self-control was a better predictor of depressive symptomatology than their Well-being, whereas for individuals with drug addiction the opposite pattern was observed. Second, while in the addiction group none of the trait EI subscales was significantly related to Social dysfunction, in the comparison group, Well-being showed a significant negative correlation. Third, in the comparison group only, Sociability was positively related to somatic symptoms. This finding would have to be replicated with the full form of the TEIQue inventory, with particular emphasis on the possibility that the assertiveness facet of trait EI Sociability may have contributed to this “maladaptive correlation” as indeed it has to others, such as that with narcissism (Petrides et al., 2011).

### Trait EI Profiles in Offspring

Children and adolescents of parents with drug addiction had similar trait EI profiles as the children and adolescents of addiction-free parents. It seems that, in this sample at least, offspring were largely unaffected by their parents’ addiction, and concomitant personality, difficulties. Possible explanations for this finding include that other people may have contributed to the development of children’s emotional functioning, including the other parent, grandparents, or friends. For instance, grandparents and friends are often reported to play a vital role in the emotional development of children, especially when parents face mental or addiction problems (Cleaver et al., 2003; Bancroft et al., 2004; Sheridan et al., 2011).

The voluntary nature of participation in this study could have led to a disproportionate recruitment of high-functioning or motivated parents. Parents with drug addiction are often apprehensive that their participation in any kind of parent–child research may lead to the removal of their children from their care (Klee, 2002). For that reason, parents who had greater relationship difficulties with their children may have refused to participate. Similarly, parents with drug-addiction, who, at the time of the research, were absorbed in themselves and their addiction difficulties, may have been indifferent to a research project seeking to improve the understanding of the parent–child emotional interaction. Clearly, such individuals would have had reduced incentives to participate.

There are no other studies specifically comparing the trait EI profiles of children of parents with and without drug addiction. However, cognate studies focusing on children’s emotional difficulties (Fals-Stewart et al., 2004) have revealed the existence of children who, despite adverse life circumstances (e.g., impaired parental mental health and parental addiction), are able to maintain an adequate level of emotional functioning and adaptive behavior. These children have been described as “resilient” (Luthar et al., 2000; Rutter, 2012; Cicchetti, 2013). In our study, children of parents with drug-addiction had similar trait EI scores as children in the comparison group. Therefore, it could be argued that these children were emotionally resilient to their parents’ addiction problems and emotional difficulties.

These results, however, should be interpreted with caution, because resilience is considered to be domain- and time-specific. A child could be emotionally resilient, but not, for instance, academically resilient (Luthar et al., 1998). Moreover, a child could be resilient at the time of the investigation, but vulnerable sometime later. Our study relied on a cross-sectional design, which cannot address whether the resilience afforded by trait EI extends from childhood to adolescence. Thus, a longitudinal design with a larger sample size is recommended for future research in order to replicate and extend our findings.

Parent and adolescent trait EI ratings were uncorrelated in both the addiction and the comparison groups. Parent and children trait EI ratings were also uncorrelated, with the exception of a significant relationship between parental Self-control and children’s global trait EI in the addiction group. It is possible that the perceived ability of parents with drug addiction to resist their impulses and regulate their stress may be conducive to the development of their children’s trait EI. Self-control is exceptionally important in individuals with drug addiction because the urge to indulge in the consumption of drugs is persistent and difficult to resist.

Previous studies on parent–offspring correlations of trait EI (Vernon et al., 2008; Gugliandolo et al., 2015) have returned moderately strong values, which were generally not observed in our samples (hypothesis H4). However, trends were in the expected direction. It is worthwhile to investigate this issue with larger sample sizes because, if confirmed, a lack of association between parent-offspring trait EI scores may be indicative of a generalized psychological break in the parent-child relationship in populations of parents with drug addiction.

### Future Research

Future research should endeavor to gather data from larger samples as well as from both parents in order to examine father-offspring, mother-offspring, and midparent-offspring correlations in drug-addiction and comparison groups. It would also be informative to examine these relationships developmentally, through a longitudinal design, with a view to enhancing our understanding of the factors that can promote or inhibit the development of trait EI in children of parents with drug addiction.

Our study examined only parents with drug addiction who participated in substitution programs. It would be desirable to extend the research to parents with drug addiction who participate in other types of programs or no programs at all. Parents with drug addiction may differ in their characteristics and priorities, depending on the type of treatment they choose. Similarly, parents with drug addiction who refuse treatment could be less motivated or more preoccupied with addiction than parents with drug addiction who participate in therapeutic programs. Finally, it would be interesting for future research to consider variables like duration of addiction and of maintenance therapy as well as dosage and tolerance of the substitution substances used.

## Conclusion

Our results lend support to the hypothesis that individuals with drug addiction have difficulties in processing and regulating emotional information. Regardless of whether these difficulties preexist or follow drug addiction, they should be treated in clinical practice and targeted in interventions. However, the finding that children in the addiction and comparison groups have similar trait EI profiles is especially important and encouraging. Previous research has revealed strong associations of trait EI with important variables like peer acceptance, pro-social behavior, socialization, body satisfaction and mental health in children and adolescents (Mavroveli et al., 2008; Frederickson et al., 2012; Resurrección et al., 2014; Gugliandolo et al., 2015; Cuesta-Zamora et al., 2018). If offspring of parents with drug addiction manage to maintain typical levels of trait EI, this would have positive repercussions in multiple other aspects of their life.

## Ethics Statement

This study was carried out in accordance with the ethical recommendations of the American Psychological Society. All subjects gave written informed consent in accordance with the Declaration of Helsinki. The protocol was approved by the Pedagogical Institution – Greek Ministry of Education, Research and Religious Affairs as well as by the Organization against Drugs.

## Author Contributions

This research was part of GA’s Ph.D. thesis at the Aristotle University of Thessaloniki. GA conceived the presented idea, developed the theoretical framework, collected the data, and wrote the first draft of the manuscript. GA and AS contributed to the design of the study. GA and KP performed the statistical analysis. KP provided feedback and critically revised the article for important intellectual content. All authors contributed to the interpretation of the results and read and approved the submitted version.

## Conflict of Interest Statement

The authors declare that the research was conducted in the absence of any commercial or financial relationships that could be construed as a potential conflict of interest.

## Footnotes

1 The Fisher z statistic was applied in all cases where both correlations were statistically significant.
